# Distribution of *BoLA-DRB3* Allelic Frequencies and Identification of Two New Alleles in Iranian Buffalo Breed

**DOI:** 10.1100/2012/863024

**Published:** 2012-02-14

**Authors:** J. Mosafer, M. Heydarpour, E. Manshad, G. Russell, G. E. Sulimova

**Affiliations:** ^1^Department of Animal Science, Ferdowsi University of Mashhad, P.O. Box 91775-1163, 9177948974 Mashhad, Iran; ^2^Population Health Research Institute (PHRI), Department of Medicine, McMaster University, Hamilton, ON, Canada L8L2X2; ^3^Moredun Research Institute, Pentlands Science Park, Midlothian, Penicuik EH26 0PZ, UK; ^4^Vavilov Institute of General Genetics, Russian Academy of Sciences, Moscow 119991, Russia

## Abstract

The role of the major histocompatibility complex (MHC) in the immune response makes it an attractive candidate gene for associations with disease resistance and susceptibility. This study describes genetic variability in the *BoLA-DRB3* in Iranian buffaloes. Heminested PCR-RFLP method was used to identify the frequency of *BoLA-DRB3* alleles. The *BoLA-DRB3* locus is highly polymorphic in the study herd (12 alleles). Almost 63.50% of the alleles were accounted for by four alleles (*BoLA-DRB3.2 ∗48*, *∗20*, *∗21*, and *obe*) in Iranian buffalo. The *DRB3.2 ∗48* allele frequency (24.20%) was higher than the others. The frequencies of the *DRB3.2 ∗20* and *DRB3.2 ∗21* are 14.52 and 14.00, respectively, and *obe* and *gbb* have a new pattern. Significant distinctions have been found between Iranian buffalo and other cattle breed studied. In the Iranian buffaloes studied alleles associated with resistance to various diseases are found.

## 1. Introduction

The major histocompatibility complex (MHC) is a large cluster of tightly linked genes that play an important role in the immune system [1, 2]. The products of these genes are involved in the induction and regulation of immune response. The MHC spans approximately 4 Mb of the human genome, 1.5 Mb in mice and approximately 2.5 Mb of the cattle genome [3, 4]. In humans, the MHC is located on chromosome 6 whereas, in cattle, it is located on chromosome 23. It has been estimated that the mammalian MHC contains over 200 genes. The structure and organization of the MHC genes of cattle, known as the bovine leukocyte antigen (*BoLA*) complex, are very similar to those of the human MHC. The genes are organized into three distinct classes (class I, II, and III). Each of these classes is divided into regions and subregions, containing pseudo genes. The major difference between the organizations of the *BoLA* complex that of the human MHC and the *BoLA* complex is that found in two separate regions of the chromosome rather than a single cluster of genes seen in most mammals. The larger gene cluster is located at BTA23 band 22 and apparently contains all of the bovine class I and class III sequences and genes encoding both subunits of the classical class II proteins *DQ* and *DR*. The remaining *BoLA* class II loci (*DIB*, *DNA*, *DOB*, *DYA*, *DYB*, *TCP1*, *LMP2*, *LMP7*, and *TAP2*) are located in a cluster near the centromere at BTA23 band 12-13 [4]. Comparative analysis suggests that the disruption was likely caused by a single chromosomal inversion.

Of the class II genes, cattle express one *DR* gene pair (*DRA* and *DRB3*) and one or two *DQ* gene pairs per haplotype. The coding sequence of *DRA* is monomorphic, while the *DRB3* gene has over 103 identified alleles [5]. As a result of this polymorphism as well as its functional importance, the *DRB3* locus and its gene products are among the best defined in cattle. Associations between *BoLA* alleles and disease have also been identified for class I and class II genes. Class I associations include tick resistance [6], nematode egg worm counts [7, 8], resistance to persistent lymphocytosis caused by bovine leukemia virus (BLV) [9], chronic posterior spinal paresis (PSP) [10], Ketosis [11], resistance to dermatophilosis [12] and mastitis [11, 13–15]. Associations have been demonstrated between genes in this region and diseases such as decreased risk of cystic ovarian disease and retained placenta [16], resistance to persistent lymphocytosis caused by BLV [17], and mastitis [15, 16, 18]. Raising buffalo has changed from a novelty enterprise to a profitable one. Buffalo may be marketed for their meat and byproducts, for recreational hunting, and breeding stock. Research on buffalo is also important because buffaloes have a higher tolerance to cold temperatures than domestic cattle and therefore exhibit greater winter hardiness. Buffalos superior digestion of low-quality feeds also makes it better suited for production on marginal rangelands. Buffaloe live longer than domestic cattle. Buffalo cows remain productive until 20 years of age. The typical replacement rate for buffalo cows is 10 percent. Cows can be bred to calve at three years of age. The demand for buffalo meat has increased primarily because consumers perceive that it has less intramuscular than beef and pork. Some also believe that this means the meat is lower in cholesterol, though this has not been proven. Some consumers prefer the taste of buffalo over the taste of beef or pork. Other valuable byproducts from buffalo include mounted heads, skulls, and hides. In this study we analyzed the polymorphism of *BOLA-DRB3* gene in Iranian Mazandarani Buffalo using PCR-RFLP, in order to identify alleles of the gene. 

## 2. Materials and Methods

### 2.1. DNA Isolation

Iranian Mazandarani Buffalo (*n* = 100) from Miankaleh Island in north of Behshahr from Mazandaran city were used in this study. Approximately 5–10 mL of blood was collected from each animal via the jugular or mammary vein, and aliquots of the whole blood were stored at −20°C. The DNA was isolated from the whole blood by a modified InstaGene protocol (Bio-Rad, Melville, NY). Briefly, the whole blood (0.3 mL) was washed once with 1 mL of PBS buffer, pH 7.2. InstaGene purification matrix (0.2 mL; Bio-Rad) was added to the pellet. The mixture was vortexed and incubated at 56°C for 60 min, followed by 15 min incubation at 99°C. The cell lysate was than centrifuged for 5 min at 7000 ×g. From the supernatant, 0.175 mL was withdrawn and diluted two-fold with 10x magnesium-free thermophilic buffer (500 Mm KCL, 100 mM Tris-HCL (PH 9.0), and 1% Triton x-100), and 2 *μ*L of this mixture was used for DNA amplification.

### 2.2. BoLA-DRB3.2 Gene Amplification

DNA amplification of the *BoLA-DRB3.2* gene was achieved by a two-step PCR, using primers and methods designed for the *BoLA-DRB3* locus. The oligonucleotide primers HL030 (5′-ATCCTCTCTCTGCAGCACATTTCC-3′), HL031 (5′- TTTAATTCGCGCTCACCTCGCCGCT-3′), and HL032 (5′- TCGCCGCTGCACAGTGAAACTCTC-3′) were used. Reaction 1 was carried out in a total volume of 25 *μ*L containing 200 *μ*L of each dNTP, 0.5 *μ*m of each primer of HL030 and HL031, 2.5 *μ*L of 10x magnesium-free thermophilic buffer (500 mm KCL, 100 mm Tris-HCL (PH 9.0), and 1% Triton X-100), 2.5 *μ*L of 25 mm MgCl_2_, 13.5 *μ*L of sterile H_2_O, 5U of Taq DNA polymerase (Promega, San Diego, Calif) and 2 *μ*L of genomic DNA. Each reaction mixture was overlaid with 20 *μ*L of mineral oil. Following an initial denaturation step at 94.5°C for 270 s, the parameters for the thermocycler (Ericomp, San Diego) were set at 94.5°C for 90 s, 66°C for 120 s, and 72°C for 60 s. Ten cycles of DNA amplification were performed followed by one step of 72°C for 5 min. From this DNA amplification reaction mixture, 4 *μ*L was used for the second PCR reaction. The second reaction was carried out in a total volume of 100 *μ*L containing 200 *μ*m of each dNTP, 0.5 *μ*m of each primer of HL030 and HL032, 10 *μ*L of 10x magnesium-free thermophilic buffer, 10 *μ*L of 25 mM MgCl2, 59 *μ*L of sterile H_2_O, and 15 U of Taq DNA polymerase (Promega). Each reaction mixture was overlaid with 20 *μ*L of mineral oil. Parameters for the thermocycler were set at 94.5°C for 90 s, 66°C for 30 s, for a total of 30 cycles, followed by a step of 72°C for 5 min.

### 2.3. Restriction Endonuclease Digestion

The PCR-amplified DNA fragments from the second PCR restriction were digested with restriction endonuclease *Rsa*I, *BstYI*, and *Hae*III (New England Biolabs, Beverly, Mass). For the restriction endonuclease digestion restriction, 10 *μ*L of the second PCR restriction product was used for each digestion. Samples were digested with *Rsa*I or *Hae*III (20 units, New England Biolabs) for 2 h at 37°C in a total volume of 20 *μ*L. Samples were digested with *BstYI* (20 units, New England Biolabs) for 2 h at 50°C followed by 15 min at 85°C in a total volume of 20 *μ*L.

### 2.4. Agarose Gel Electrophoresis

Restriction enzyme digested samples were electrophoresed in 4% agarose (Metaphor; FMC Bioproducts, Rockland, Me) with TBE buffer (0.9 m tris base, 0.09 m boric acid, and 2.5 mm EDTA; pH 8.3). Gels were run at 100 V for 4 h and stained with ethidium bromide (1.0 *μ*g l mL; Sigma Chemical Co., St. Louis, Mo). The DNA was visualized by transillumination (Foto dyne Inc., New Berlin, Wis) and photographed with type 55 polaroid film (Polaroid Corp., Cambridge, Mass). The negative of the type 55 polaroid film was scanned and analyzed with gel manager for windows (Biosystematica, Devon, UK). The *BoLA-DRB3.2* PCR-RFLP nomenclature was used for *BoLA-DRB3* exon 2 alleles as described by [19] and maintained in the BoLA immunopolymorphism database (http://www.ebi.ac.uk/ipd/mhc/bola/). New or unpublished allele types were identified using the restriction enzyme pattern as described by [19]. 

### 2.5. Sequence Analysis

Sequencing of new alleles was done by chain termination method on ABI 377 sequencing machine (GNTC Co., Germany). Sequencing results were analyzed using FASTA program, and the sequencing was compared against the other alleles by using BLAST at NCBI site (http://www.ncbi.nlm.nih.gov/). 

## 3. Results

**Figure 1:**
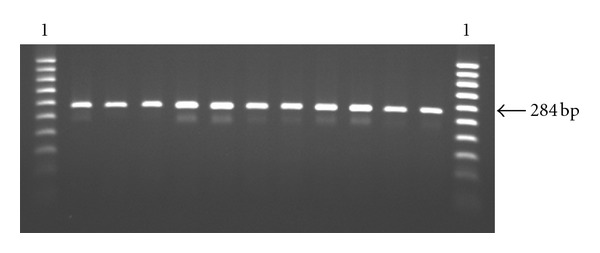
Heminested PCR products. Lane 1 is 50 bp molecular marker. The other lanes are PCRproducts of *BoLA-DRB3.2* with 284 bp size.

**Figure 2:**
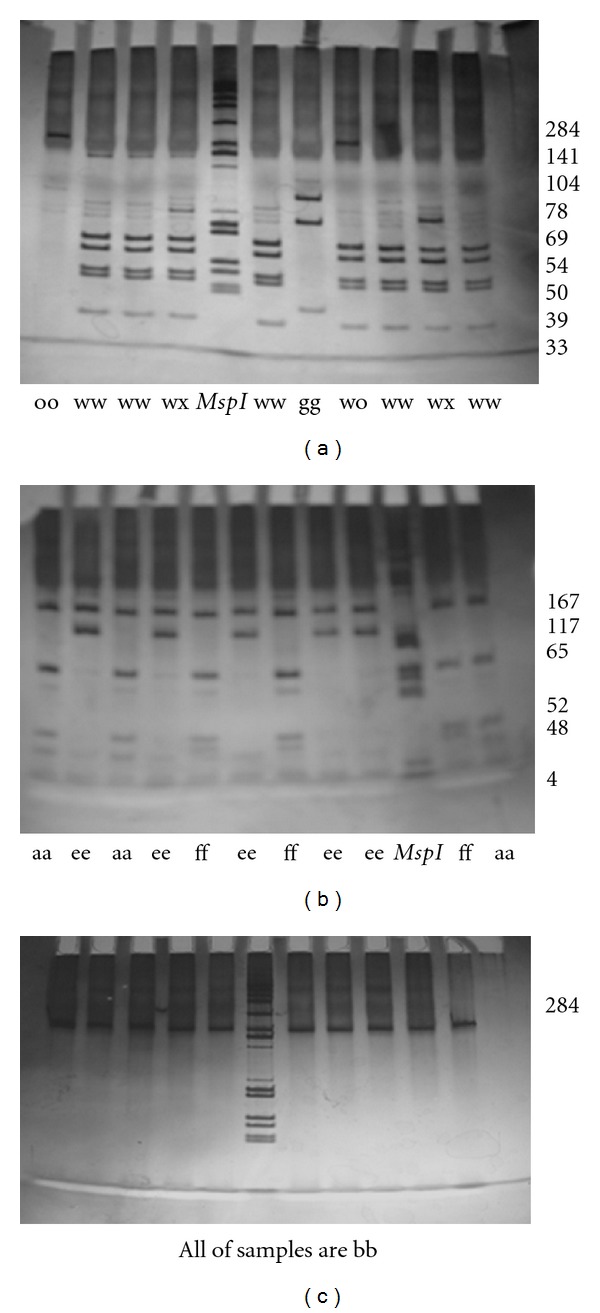
Electrophoresis in 8% polyacrylamide gel of exon 2 amplification products of gene *BoLA-DRB3 *digested by endonucleases *Rsa*I (b), *Hae*III (a), and *BstYI* (c). *Msp*I fragments of plasmid pUC19 are used as a molecular marker. The length of fragments composing *Rsa*I, *Hae*III, or *Bst*YI DNA patterns is shown on pictures.

We used heminested PCR-RFLP method for identification of the frequency of *BoLA-DRB3* alleles in Iranian Mazandarani Buffaloes. PCR products were represented by 284 bp fragments as was expected on the basis of the nucleotide sequence of the gene (Figure 1). The spectra of *Rsa*I, *Hae*III, and *BstYI *restriction sites are shown by [19]. Typical patterns of restriction of the amplified products with the endonucleases *Rsa*I, *Hae*III, and *BstYI* are shown in Figure 2. This is the first study of the DNA polymorphism of the *BoLA-DRB3* gene in Iranian buffaloes. We can demonstrate that the *BoLA-DRB3* locus is highly polymorphic in the studied herd. The *BoLA-DRB3* in Iranian Mazandarani Buffalo has 12 alleles (Table 1). Analyzing *BoLA-DRB3* of periparturient buffalo reported finding 12 allele types. More significant distinctions have been found between Iranian buffaloes and cattle breeds studied. Almost 63.50% of the alleles were accounted for by four alleles (BoLA-DRB3.2 *48, *20, *21, and* obe*) in Iranian Mazandarani Buffaloes. The frequencies of the DRB3.2 *43, *50 and *gbb* alleles were lower than the other alleles in this breed. In addition to these alleles we found two new alleles. The nucleotide sequences of the amplified exon 2 of these alleles were determined and submitted in the GenBank database under accession no. DQ187336 for *obe* allele and DQ187337 for *gbb* allele. Afterwards, they were compared to the *DRB3 *alleles published by the *BoLA *Nomenclature Committee using the program BLAST from National Center for Biotechnology Information (http://www.ncbi.nih.gov/).

## 4. Discussion

This is the first study of the DNA polymorphism of the *BoLA-DRB3.2* gene in Iranian Mazandarani Buffalo. Previous work in *Bubalus bubalis* defined 8 *DRB* alleles among 75 animals from different buffalo populations in different countries [20], while a study of 25 unrelated Indian river buffalo revealed 22 *DRB* alleles [21]. These data, together with the present study, demonstrate that allelic frequencies of *BoLA-DRB3 *appear to depend on the breed and population, likely a result of founder population structure and selection pressure. Our present study demonstrated that the *BoLA-DRB3* exon 2 is highly polymorphic in Iranian Mazandarani Buffalo. We found 12 PCR variants in Mazandarani Buffalo. The most frequently isolated alleles in our buffalo were BoLA-DRB3.2 *48, *20, *21, *obe*, *19, *13, *42 and these accounted for about 81% of the alleles in this population. But the alleles that Sena reported were not found in this study on Mazandarani Buffalo. The polymorphism of *BoLA-DRB3* by DNA sequence analyses of 18 African cattle investigated by [22]. They found 18 alleles was in small random sample of animals from two breeds. Then they found 30 alleles in *B. P. taurus* cattle in Africa and Zebu cattle [23]. A research on 127 Brahman cattle (zebu) in Martinique said that an amino acid sequence coded by the exon 2 of *BoLA-DRB3* gene associated with a *BoLA* class I specificity constitutes a likely genetic marker of resistance to dermatophilosis [12]. 13 alleles within eight major allelic families in African *Bos indicus* and *Bos Taurus* cattle were found by [23]. They found 7 alleles from *DQA3* within three major allelic families. 18 alleles in a research on 568 zebu Brahman cattle (*Bos indicus*) from Martinique (French West Indies) in *BoLA-DRB3* exon 2 were found by [12]. They said that 5 official alleles DRB3.2 *0301, *0302, *0901, *0902, and *1202 correlate with the susceptibility. They found another correlation between susceptibility and the BoLA-DQB *1804 allele. A research on zebu Brahman cattle in Martinique (FWI) showed that MHC molecules can control diseases such as dermatophilosis [12].

There are some articles about *BoLA-DRB3.2* in other cattle. Polymorphism of the *BoLA-DRB3* gene has been reported in the studies of Jersey [24]. This breed has a different allele and allele frequency profile from buffaloes. For example, the six most frequently detected alleles in Jersey cows are BoLA-DRB3.2 *8, *10, *15, *21, *36, and *ibe*, accounting for approximately 74% of the alleles in the population of the herd (172 animals). But the six most frequently detected alleles (BoA-DRB3.2 *8, *9, *21, *27, *7, and  *24) accounted for 70% of the alleles in a population of Japanese shorthorn cows [5]. Between these alleles only we saw BoLA-DRB3.2 *21 on the Mazandarani Buffalo; therefore we can say that alleles and their frequency were different between buffaloes and other breeds of cattle. In Argentine Creole cows (194 animals) six most frequently detected alleles (BoLA-DRB3.2 *15, *18, *24, *20, *27, and  *5) that account for approximately 73% of the alleles in the herd and BoLA-DRB3.2 *20 were found in Iranian Mazandarani Buffalo. In Russian Ayrshire cattle allele DRB3.2 *7 is prevalent by 37.6%, and the combined frequency of alleles DRB3.2 *7, *28, *10, and *24 is 77% [19]. Alleles *22, *24, *11, *16, *18, *23, *8, and *27 are the most frequent in Russian black pied cattle that were not found in Mazandarani Buffalo in this research [25]. In a study of ten breeds beef and dairy cattle were analyzed and BoLA-DRB3.2 *5, *29, and *30 alleles were identified only in south Devon, Angus, Gelbvieh and the BoLA-DRB3.2 *7 allele only in Angus, Gelbvieh, and Holstein Friesian cows. Significant associations have been made between *BoLA* genes and some infection diseases of cattle, particularly diseases that are prevalent during lactation. For example, [15] indicated that one *BoLA-DRB3* gene pattern in a study of Holstein cows (*n* = 106) is associated with resistance to *Staphylococcus aureus* mastitis. Three alleles (DRB3.2 *11, *23, and *28) determine resistance to leukemia and four (DRB3.2 *8, *16, *22, and *24) are associated with susceptibility.

There was a lot research on *BoLA-DRB3.2* in Iranian cattle (Table 2). For example, [26] found 15 alleles in Iranian Sarabi cattle. Their frequency was between 2 and 23%, and 4 of these alleles (BoLA-DRB3.2 *48, *43, *20, and *19) were found in Iranian Mazandarani Buffaloes in this study. They found 16 alleles in Iranian Najdi cattle that have 2–13% frequency and 1 of them (BoLA-DRB3.2 *43) was found in Iranian Mazandarani Buffalo. 19 alleles in Iranian Sistani cattle were found by [27] that have 1–22% frequency and 3 of them (BoLA-DRB3.2 *37, *21, and *13) were found in Iranian Mazandarani Buffalo. In addition, [28] found 19 alleles were found in Iranian Golpayegani cattle that have 2–14% frequency and 2 of them (BoLA-DRB3.2 *20, and *19) were found in Iranian Mazandarani Buffalo. Also, [29] found 26 alleles in Iranian Holstein cattle that have 2–26.6% frequency and 5 of these alleles (BoLA-DRB3.2 *49, *37, *21, *20, and *13) were found in Iranian Mazandarani Buffalo. In this study we found that our data indicate that allelic frequencies of *BoLA-DRB3* may, at least to some extent, depend on the breed and geographical location. Similarities between the immune systems of cattle and buffalo and the overlapping range of pathogens that infect these two livestock species may support the view that disease associations discovered for the *BoLA* system can have practical application in buffalo. Continued analysis of *BoLA-DRB3 *alleles in large agricultural populations may help reduce the spreading of alleles providing susceptibility to diseases such as mastitis or leukemia in buffalo herds. Thus, investigation of polymorphism of the *BoLA-DRB3 *gene may have great practical as well as theoretical value.

## Figures and Tables

**Table 1:** Frequencies of *BoLA*-*DRB3.2* alleles in Iranian Mazandarani Buffalo (*n* = 100) as identified by polymerase chain reaction and restriction fragment length polymorphism analysis. Corresponding DNA patterns are shown at the bottom of the figures. Nomenclature of DNA patters is given corresponding to http://www.projects.roslin.ac.uk/bola/drb3pcr.html. Standard errors of the allele frequencies do not exceed 5%.

Alleles	DRB3 PCR-RFLP	* Rsa*I/*BstYI*/ *Hae*III patterns	Frequencies (%)
DRB3 *3901	*48	Wba	24.20
DRB3 *2301, *2901, *3601	*20	Ibb	14.52
DRB3 *0801	*21	Ibe	14.00
*New *	new	Obe	10.75
DRB3 *2601	*19	Sbb	10.22
DRB3 *0401	*13	Hba	9.14
DRB3 *2802	*42	Hbf	8.51
DRB3 *3701	*49	Wbe	2.70
*DRB 07 *	*37	Oba	2.70
DRB3 *25012	*43	Kbf	1.08
DRB3 *4001	*50	Xba	1.08
*New*	new	gbb	1.08

**Table 2:** Frequencies of *BoLA-DRB3.2 *alleles for the studied Iranian cattle breeds.

*DRB3 *alleles	Sarabi (*N* = 52)	Najdi (*N* = 52)	Sistani (*N* = 49)	Golpayegani (*N* = 50)
2	10	2	—	4
3	2	2	1	2
4	—	—	2	3
7	—	—	4	11
8	2	8	**22 **	—
10	—	—	4	6
11	18	11	5	7
12	10	5	—	6
13	—	—	3	—
14	2	**13 **	—	—
15	—	5	8	2
16	—	—	—	**14 **
17	2	2	—	—
19	2	—	—	10
20	2	—	—	2
21	—	—	2	—
22	2	2	—	2
23	15	11	—	—
24	—	**13 **	2	2
25	3	—	—	2
28	—	—	—	8
29	—	—	1	—
31	—	—	—	4
34	—	—	21	—
35	—	—	—	3
36	—	11	2	—
37	—	—	1	—
43	6	3	—	—
44	—	—	6	—
45	—	—	2	6
47	—	—	3	—
48	5	—	—	—
51	—	—	1	—
52	**23 **	6	—	6
53	—	5	—	—
54	—	5	—	—
*X*	—	—	8	—
